# The Reactivity of Hydroxyl Radicals toward Boric Acid as a Function of pH

**DOI:** 10.1021/acs.jpca.4c03933

**Published:** 2024-09-03

**Authors:** Fredrik Petersson, Mats Jonsson

**Affiliations:** Department of Chemistry, KTH Royal Institute of Technology, SE-100 44 Stockholm, Sweden

## Abstract

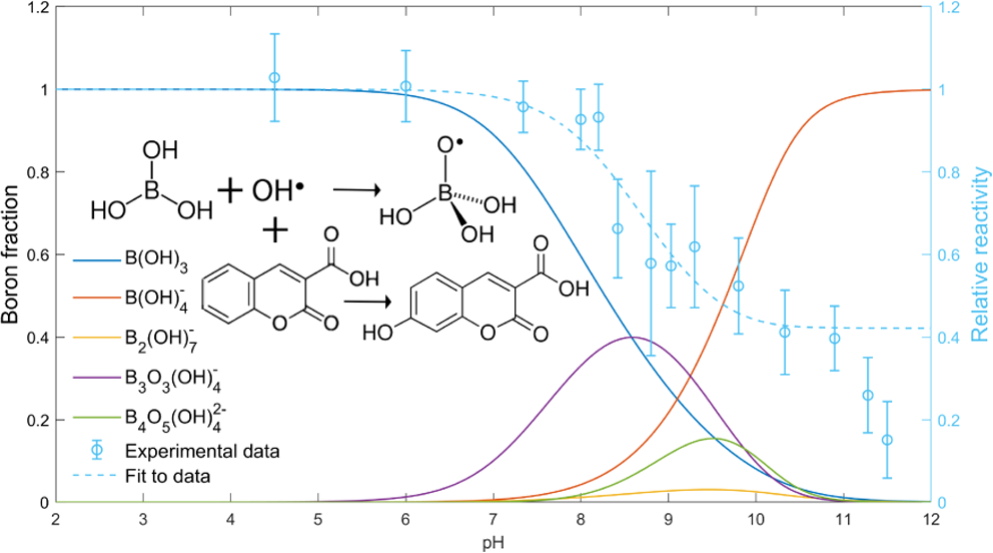

Boric acid and its counter-base, borate, are a commonly used buffer pair in many systems where hydroxyl radicals are generated. Boric acid is also used in light water-cooled nuclear reactors to control the excess reactivity of the nuclear fuel. Hydroxyl radicals are generated within the cooling water of the reactor because of intense radiation. The reactivity of the hydroxyl radical toward boric acid has previously been studied, but to the best of our knowledge, only upper limits of the rate constants are available in the literature. In this study, the rate constants for the reaction between the hydroxyl radical and boric acid and its counter-base including several polyborates that form at high boron concentration are determined. The rate constants were determined from competition kinetics using steady-state γ radiolysis and coumarin-3-carboxylic acid as the competing reactant. By varying the pH and accounting for boron speciation, it was possible to determine the rate constant for the different boron species using multilinear regression. The rate constants for boric acid and the counter-base were determined to be 3.6 × 10^4^ and 1.1 × 10^6^ M^–1^·s^–1^, respectively, which is very close to the previously determined upper limits of the rate constants. For the polyborate species diborate and tetraborate, the rate constant was determined to be 6.4 × 10^6^ and 6.8 × 10^6^ M^–1^·s^–1^, respectively.

## Introduction

To increase the uptime and reduce the downtime of nuclear power stations, nuclear reactors are nowadays loaded with more fissile material than in the past.^1−3^ This leads to longer fuel cycles. However, the additional fissile material will lead to an excess of reactivity at the beginning of the fuel cycle. To compensate for the initial excess reactivity, burnable neutron absorbers are added. The most common way to introduce a neutron absorber in the reactor is by using control rods, but burnable poison mixed within the fuel in some rods or absorber dissolved in the coolant water are also common solutions.^4^ Usually, a combination of all three is used. The disadvantage of the two heterogeneous methods of absorbing neutrons is the more uneven reactivity control compared to homogeneously dissolved neutron absorbers; the more unevenly distributed reactivity control can lead to a shift in power distribution. Another drawback of having many control rods is that they are expensive. The advantage of a dissolved neutron absorber is its homogeneous distribution within the reactor core. Boric acid is widely used as a dissolved neutron absorber in pressurized water reactors but not in boiling water reactors due to the risk of precipitation on surfaces where steam forms.^5^ When utilizing higher fuel burnup, the initial concentration of boric acid must be higher than for a conventional burnup fuel cycle.^6^ Some design concepts of small modular reactors based on the pressurized water reactor, PWR, are designed to operate without boric acid as reactivity control and only use control rods and burnable poison mixed in the fuel rods.^7^ This is to make the reactor simpler and to remove some auxiliary systems.

Boron is used in PWRs since Boron-10 has a high cross section for thermal neutrons. It is used in the form of boric acid because of its high solubility. When Boron-10 captures a neutron, an α particle and a Lithium-7 ion will be formed. These two particles will have excess energy in the form of kinetic energy. The particles are classified as heavy charged particles capable of ionizing materials that they traverse. In general, heavy charged particles have high LET-values (linear energy transfer), which means that ionizations and excitations are dense, resulting in a very short penetration depth. This also has implications on the water chemistry in the reactor since dense ionizations lead to high local concentrations of radicals which in turn favors radical–radical combination yielding molecular products like H_2_O_2_, H_2_, and O_2_ through radiolysis.^8^ At the start of the fuel cycle, when the reactor has the maximum content of fissile material, the concentration of boric acid will be the highest. Throughout the fuel cycle, the boron content is reduced along with the burning of the fuel.^6^ The concentration of boric acid at the start of the fuel cycle is around 2000 ppm (30 mM) and decreased to 0 at the end. The pH in the reactor under operation is between 6.9 and 7.5, and the temperature is 300 °C. Boric acid is a very weak acid with a p*K*_a_ of 9.24 at room temperature.^9^ The counter-base is the borate ion, and it will be present to some extent in the nuclear reactor. At high boric acid concentrations, polyborates are also formed, the most common are diborate, triborate, and tetraborate.^10,11^ The polyborates are formed at a pH around the p*K*_a_ of boric acid and disappear with increasing pH. For temperatures up to 150 °C, the triborate is the dominating species of the polyborates. From temperatures above 200 °C, the diborate is the dominating one.^12^

In water-cooled nuclear reactors, the water is continuously exposed to intense radiation from γ photons, neutrons, and heavy charged particles originating from neutron capture by Boron-10 in case the reactor is operated with dissolved Boron-10. In general, radiolysis of water produces e_aq_^–^, H, H_2_, HO, HO_2_ and H_2_O_2_ as primary products and O_2_ as a secondary product. These species are all redox-active and can contribute to the corrosion of reactor materials. The rate at which this occurs is strongly dependent on the presence of other solutes in the cooling water. For this reason, the reactivity of boric acid and its counter-base toward aqueous radiolysis products is of prime interest.

Boric acid and borate are used in more places than in nuclear reactors. It is also a very common buffer pair to control the pH.^13^ The borate buffer is used in many systems, where reactions with hydroxyl radicals are investigated because it is regarded as being inert. The method of hydroxyl radical generation in the boron-containing systems has been mainly pulse radiolysis but also steady-state γ radiolysis and photolysis.^14−21^ Borate is commonly used in the range of 1–10 mM total boron concentration to set the pH between 8 and 10. The reaction of boric acid, borate, and also polyborates, if the concentration is high enough, toward the hydroxyl radical is something that needs more data to give a better insight into the reaction kinetics.

The reactivity of boric acid toward the hydroxyl radical has previously been studied both from photolytically generated hydroxyl radicals, pulse radiolysis, and steady-state γ radiolysis.^22−25^ The first study from Ohno in 1967 produced hydroxyl radicals from nitrous oxide reacting with hydrated electrons produced from ferrocyanide solutions illuminated at a wavelength of 253.7nm. It was concluded that the rate constant for the reaction with the hydroxyl radical was 1.9% of that for the reaction between ferrocyanide and the hydroxyl radical (1 × 10^10^ M^–1^·s^–1^).^24^ In a later study by Buxton and Sellers,^22^ the reaction was investigated by both pulse radiolysis and steady-state γ radiolysis, but no reaction could be confirmed. However, it was concluded that the upper limit of the rate constant was 5 × 10^4^ M^–1^·s^–1^. For the counter-base of boric acid, an upper limit for the rate constant of the reaction with the hydroxyl radical was determined as 1 × 10^6^ M^–1^·s^–1^.^26^

Monitoring the hydroxyl radical in real time is not straightforward. A common way to circumvent this difficulty when studying the reactivity of the hydroxyl radical is to use competition kinetics. To do this, a reference reaction is required where it is possible to monitor either the consumption of reactant or the formation of product. For the hydroxyl radical, several different radical scavengers have been used. Some commonly used scavengers are ferrocyanide, methanol and TRIS, Tris(hydroxymethyl)aminomethane, with rate constants for the reaction with HO^**•**^ determined of 1.05 × 10^10^, 9.7 × 10^8^, and 1.1 × 10^9^ M^–1^·s^–1^, respectively.^26,27^ The two organic scavengers form formaldehyde which can be determined spectrophotometrically using the so-called Hantzsch method.^28^ However, fairly recent studies have shown that formaldehyde is not a unique product for the hydroxyl radical.^29,30^ The reaction between ferrocyanide and the hydroxyl radical oxidizes ferrocyanide to ferricyanide, which can be measured directly at a wavelength of 420 nm.^31^ Another probe which has been used is coumarin-3-carboxylic acid, 3CCA.^32−34^ The hydroxyl radical reacts with coumarin-3-carboxylic acid through an addition reaction to the most electron-rich positions of the rings (C5 and C7). The product that is hydroxylated in the C7 position is strongly fluorescent, which makes this method quite sensitive. The rate constant for the reaction with HO has been determined to be 5.0 × 10^9^ M^–1^·s^–1^.^32^ The advantage of using coumarin as a scavenger for hydroxyl radicals is that the formation of the hydroxylated product is specific to hydroxyl radicals.

Some scavengers could possibly also react with the radical formed from the reaction between boric acid and the hydroxyl radical.^35^ This could be a problem if the same product is formed as in the reaction between the scavenger and hydroxyl radical. However, it is very unlikely that the radical formed upon the reaction between boric acid and the hydroxyl radical would hydroxylate coumarin-3-carboxylic acid. From a practical point of view, it is important to point out that the yield of the hydroxylation during irradiation and the intensity of the fluorescence depends on pH, which makes it very important to control the pH during the experiments.^32^ Oxygen levels also have to be controlled during the irradiations since the yield of the fluorescent 7-hydroxycoumarin-3-carboxylic acid is increased at higher oxygen levels.^33^

**Figure 1:**
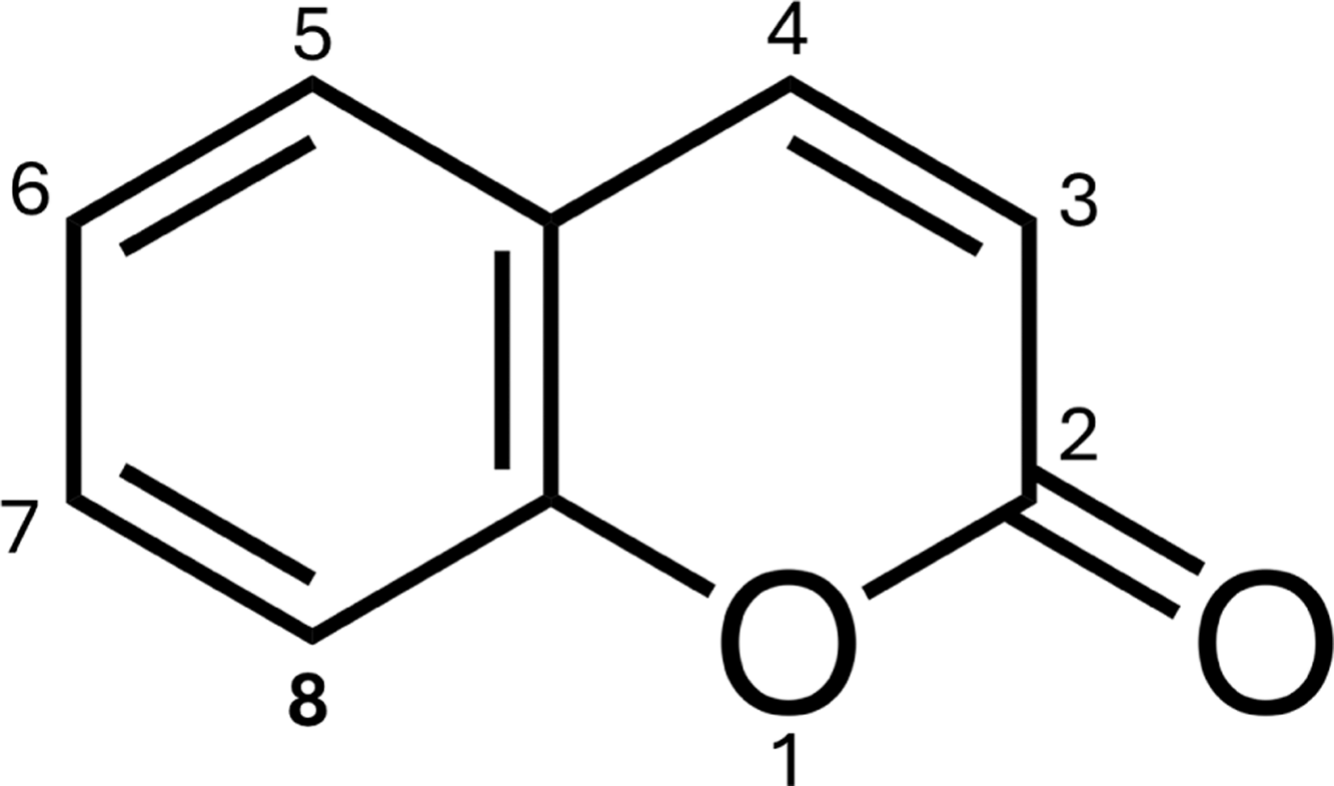
Structure of coumarin with labeled positions. Coumarin-3-carboxylic acid used in this paper has a carboxylic acid group located at the third position.

It should be pointed out that several different hydroxylated products are formed, but mainly one of them is strongly fluorescent. In Figure 1, the structure of coumarin can be seen with its positions labeled. Hydroxylation of the carbon in position 7 is the only strongly fluorescent product, this applies to both coumarin and coumarin-3-carboxylic acid.^32,36^ For the hydroxylation of coumarin, the main product is 5-hydroxycoumarin with a G-value of 23 × 10^–3^ μmol·J^–1^. The product with the second highest yield is 7-hydroxycoumarin with a G-value of 16.3 × 10^–3^ μmol·J^–1^. The 5 and 7 position are the positions with the highest yields, the other positions can be considered minor products with G-values of 1.3 × 10^–3^ μmol·J^–1^ for 3-hydroxycoumarin, 6 × 10^–3^ μmol·J^–1^ for 4-hydroxycoumarin, 7 × 10^–3^ μmol·J^–1^ for 6-hydroxycoumarin, and 2.7 × 10^–3^ μmol·J^–1^ for 8-hydroxycoumarin.^36^ The substitution pattern is expected to be similar for coumarin-3-carboxylic acid, with the exception that the third position is not available for hydroxylation because that is where the carboxylic acid group is located. It is a potential weakness of the method that 7-hydroxycoumarin-3-carboxylic acid is not the only product, since it cannot be ruled out that the relative ratio of the substitution pattern may change depending on irradiation conditions.^36^ This could be particularly relevant when comparing homogeneous systems to heterogeneous systems. In the present work, we are only dealing with homogeneous systems, and the main change in conditions is the presence or absence of the boric acid/borate. For each pH, we determine the yield of the fluorescent product with and without boric acid/borate. Hence, any possible effect of pH is canceled. As the boron-containing species is not believed to be directly involved in the hydroxylation reaction, we do not see any reason why the relative product yield should be different.

In this work, we have investigated the reactivity of HO^**•**^ toward boric acid and borate species at pH 4.5 to 11.5 using competition kinetics. Within the competition kinetic experiments, the rate constant for the reactions between and boron species is determined using the reaction of HO^**•**^ with 3CCA as a reference reaction.

## Methods

The boric acid used in the experiments was of ACS reagent grade from Sigma Aldrich. The disodium tetraborate decahydrate was of analytical grade from Merck. Coumarin-3-carboxylic acid came from Arcos Organics with 98% purity. Sodium hydroxide was provided by Merck and was of analytical grade. Water from a Milli-Q Millipore system was used for all solutions. Since the reactivity of the hydroxyl radical toward boric acid and its counter-base is expected to be low, a high concentration of boron had to be used compared to the 3CCA concentration. Solutions with a low and a high concentration of boron, 1 and 300 mM respectively, were prepared. 1 mM boron was used in the reference system to buffer the pH and make it easier to adjust the pH to the same value as in the 300 mM boron solution. The pH of the samples was adjusted with NaOH. Coumarin-3-carboxylic acid was added from a stock solution to each sample so that the concentration was 0.05 mM.

### Irradiation

Samples were irradiated at room temperature in a Cs-137 Gammacell 1000 Elite with a dose rate of 0.1 Gy/s as determined by Fricke dosimetry.^37^ 30 mL glass vials with plastic screw caps were used with 20 mL of aerated reaction solution in each. Four vials were placed in a stainless-steel holder, which, in turn, was placed in the gammacell. Since the dose rate is not homogeneously distributed inside the gammacell, the stainless-steel holder was continuously rotated at a speed of 30 rpm during the irradiation. For short irradiation times, the coumarin-3-carboxylic acid concentration can be seen as constant. To avoid any large changes in concentration, the total irradiation time was selected as 8 min. Every 2 min, the vials were taken out and 0.5 mL of sample was taken from each vial; this was done four times in total.

### Fluorescence Measurements

The pH in all samples was adjusted to 9.23 with a 100 mM borate buffer solution before the fluorescence was measured. This was done because the intensity from the fluorescent product, 7-hydroxycoumarin-3-carboxylic acid, changes with pH.^32^ 25 μL of the sample was diluted to 2.5 mL using the borate buffer in a fluorescence cuvette. The fluorescence intensity was measured by using a Cary Eclipse fluorescence spectrophotometer. The excitation wavelength was set to 385 nm, and the emission was measured at 450 nm. The excitation and emission slit widths were both set to 10 nm, and the average signal time used was 5 s.

## Results and Discussion

**Figure 2:**
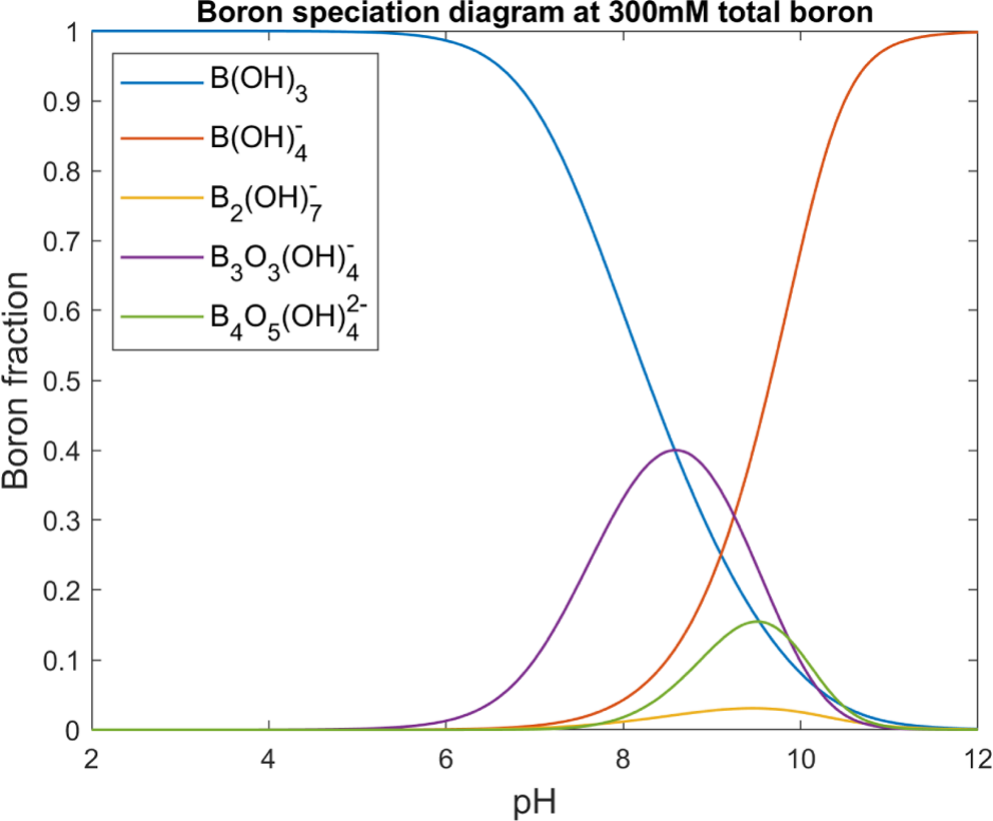
Fraction diagram of 300 mM boric acid for a pH range of 2–12 and a temperature of 25 °C. The five species included in the diagram are boric acid, (B(OH)_3_), borate, (B(OH)_4_^–^), diborate, (B_2_(OH)_7_^–^), triborate, (B_3_O_3_(OH)_4_^–^), and tetraborate, (B_4_O_5_(OH)_4_^2–^).

As mentioned above, boron speciation can be quite complex at a higher pH. Boric acid acts as a Lewis base and reacts with water to form the borate ion. Boric acid can also form several polyborate species. The most common are dimers, cyclic trimers, and tetramers.^13^ The distribution between boric acid, borate, and the polyborate species will change depending on pH and total boron concentration. At a low pH, boric acid is the dominant form, while at a high pH, borate will dominate. Most polyborate species form at pH around the p*K*_a_ of boric acid and at high total boron concentrations. The equilibrium reactions for the most common boric acid and borate species can be seen in reactions 1–4. The logarithm of the equilibrium constants, log K, for the reactions at 25 °C are 9.234, 9.35, 7.326, and 16.23, respectively.^10,12^ The distribution of boron species was calculated using Medusa.^38,39^Figure 2 shows the fraction diagram of boron at a total boron concentration of 300 mM. The equilibrium constants are temperature-dependent, and for the pH range where polyborates form, i.e., close to the p*K*_a_ of boric acid, the most dominant species is the triborate for temperatures up to 150 °C. At temperatures above 200 °C, the diborate is the dominant species.^12^
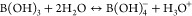
1
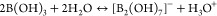
2
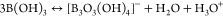
3
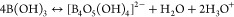
4

As stated above, the yield of 7-hydroxycoumarin-3-carboxylic acid is dependent on pH. The fluorescence intensity and the maximum excitation wavelength are also pH-dependent, making it very important to control the pH. The hydroxylated product has a p*K*_a_ of 7.4 and is mainly excited at two bands, one at 345–365 nm and another at 385–395 nm.^32−34^ When measuring at a pH lower than the p*K*_a_, the largest part of the excitation comes from the lower band, and at pH above the p*K*_a_, the excitation mainly occurs at the upper band. The emission wavelength is not dependent on pH and the maximum emission is constant at 450 nm.^32^ For the irradiations that were performed at a pH lower than the p*K*_a_ for boric acid, boric acid was used and the pH was increased by adding NaOH. For irradiations performed at pH above the p*K*_a_, sodium tetraborate decahydrate was used instead, which will dissociate into boric acid and the borate ion. All measurements were made at an excitation wavelength of 385 nm, because it was where the maximum excitation was at a pH above 7.4. Excitation and emission spectra of 7-OHCCA at pH 9.23 can be seen in Figure S1. In a continuously irradiated system, like this, the concentration of hydroxyl radicals rapidly reaches steady state. At steady state, the rate of radiolytic hydroxyl radical production is balanced by the rate of hydroxyl radical consumption. At low dose rates, the concentrations of radicals will be low, which reduces the impact of radical–radical reactions. The rate of hydroxyl radical consumption will therefore be given by *k*_3CCA_ × [HO^**•**^] × [3CCA] + [HO^**•**^] × ∑(*k*_Bn_ × [*B*_n_]) in a system containing both the boron species and the coumarin-3-carboxylic acid. [B_n_] is equal to the boron concentration for each species and *k*_Bn_ is the rate constant for the corresponding boron species. The use of summation of B_n_ species is due to the coexistence of several boron species. The fraction giving rise to the fluorescent product is given by . This fraction can also be written as , where [*P*]_i_ is the product concentration in a system containing both solutes and [*P*]_ref_ is the product concentration in the reference system only containing 3CCA. Eq 5 illustrates this relationship. By inverting this expression, we obtain eq 6 from which *k*_Bn_ can be determined.
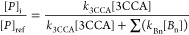
5
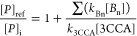
6

To achieve reasonable sensitivity in the competition kinetics, it is preferable to have [*P*]_i_/[*P*]_ref_ in the range of 0.1–0.9. As the reactivity toward boric acid is expected to be low,^22,23^ the boric acid concentration must be much higher than the 3CCA concentration. In the following experiments, the concentration of 3CCA was 0.05 mM and the total boron concentration was 6000 times higher, 300 mM.

**Figure 3:**
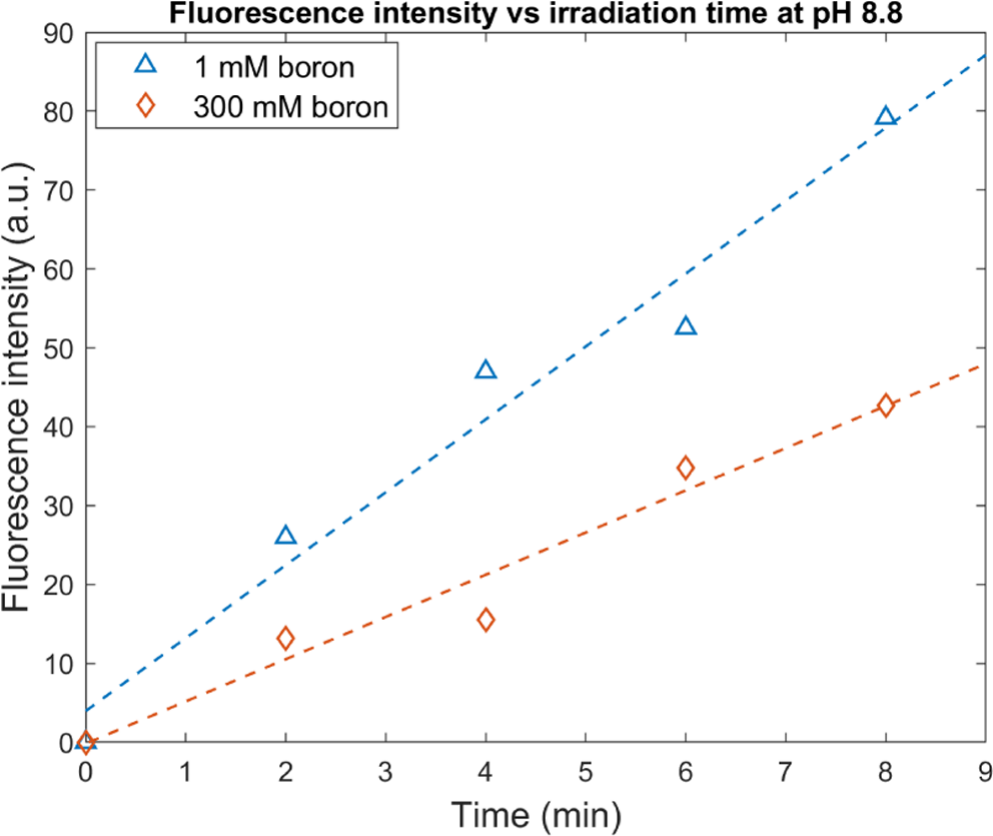
Fluorescence intensity at 450 nm using an excitation light of 385 nm for one sample with a low total boron concentration of 1 mM and one sample with a high boron concentration of 300 mM. The two samples were irradiated at pH 8.8, and the fluorescence intensity was measured at a pH of 9.23. The slopes of the fluorescence intensity are 9.24 ± 0.97 and 5.35 ± 0.63 for the 1 mM boron solution and the 300 mM boron solution, respectively.

Irradiations were performed in the pH range of 4.5–11.5. To better control the pH, a small amount of boric acid or tetraborate, 1 mM of total boron, was added to the reference sample containing only 3CCA. Since the rate constant for the reaction between hydroxyl radicals and boric acid or borate is expected to be very low, 1 mM boron is not expected to effectively compete with 3CCA. In Figure 3, the fluorescence intensity from 7-OHCCA is plotted as a function of irradiation time at pH 8.8. The two solutions were irradiated at the same time, and it is apparent that the yield is higher from the 3CCA solution with a low boron concentration. In the solution containing 300 mM boron, the formation of 7-OHCCA is inhibited due to the competition. The slopes of the fluorescence intensity as a function of the irradiation time are 9.24 and 5.35 for the low and high boron concentrations, respectively. This corresponds to a [P]_i_/[P]_ref_ ratio of 0.58. Figures of fluorescence intensity versus irradiation time for pH 4.5–11.5 can be seen in Figures S2–S15.

**Figure 4:**
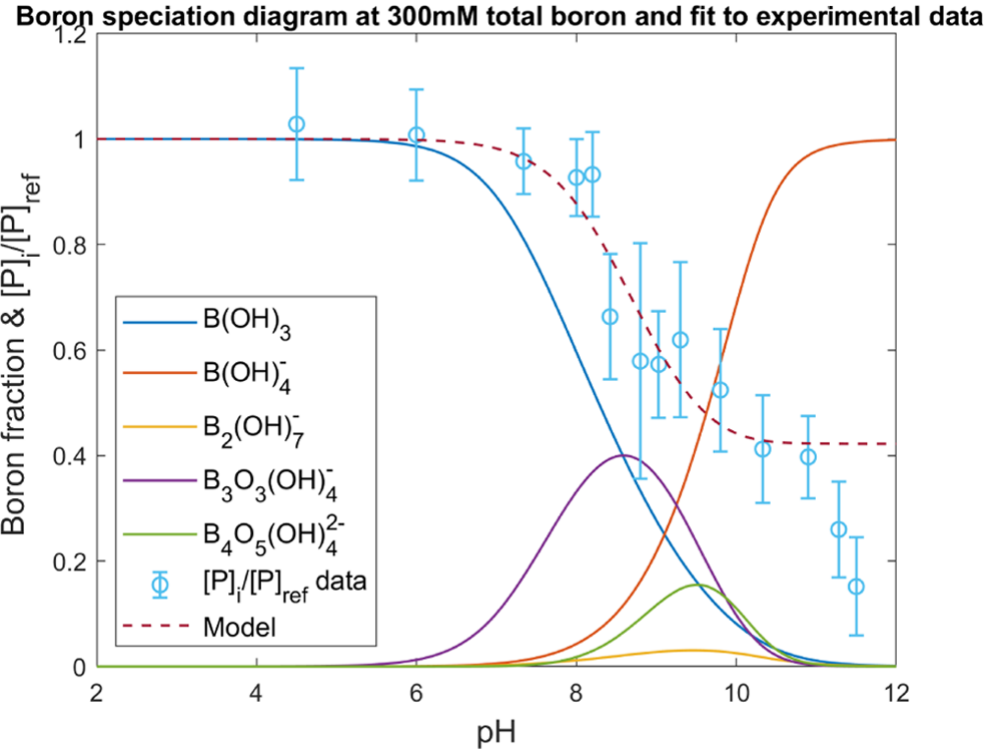
Boron speciation diagram overlaid the experimental data of the [*P*]_i_/[*P*]_ref_ ratios and the model fitted to the experimental data. The deviation from the model by the measurements at pH values above 11 can be explained by the deprotonation of the hydroxyl radical with a p*K*_a_ of 11.9 and the presence of carbonate as an impurity from sodium hydroxide.

The combination of the results from all of the irradiations within the pH interval can be seen in Figure 4 where the ratio [*P*]_i_/[*P*]_ref_ from the experimental data is plotted as circles against the pH. No competition is observed in the area where only boric acid is occurring i.e., pH < 6. The yield of 7OHCCA is unchanged between the high and low concentrations of boron at pH 4.5 and 6. However, this does not mean that the hydroxyl radical is unreactive toward boric acid. An upper limit of 5 × 10^4^ M^–1^·s^–1^ for the reaction was previously estimated by Buxton and Sellers.^22^ Using eq 6, this rate constant would give a [*P*]_i_/[*P*]_ref_ ratio of 0.94. Most probably, the reaction is even slower than this since the ratio of the yields is even smaller.

When increasing the pH to 7 and above under the conditions described above, the yield of the hydroxylated product is reduced, which indicates that the reaction between the hydroxyl radical and the boron species starts to become competitive. Under these conditions, several boron species are present (see Figure 2 or 4). These are mainly the borate ion B(OH)_4_^–^, diborate B_2_(OH)_7_^–^, triborate B_3_O_3_(OH)_4_^–^, and tetraborate B_4_O_5_(OH)_4_^2–^. The reactivity of the borate ion was previously estimated to have an upper limit of 1 × 10^6^ M^–1^·s^–1^,^26^ but no values of diborate, tetraborate, and triborate have been found in the literature.

Boric acid and borate are the only boron species that exist alone in the system at low or very high pH. The other three species are present in mixtures of only two or more species. To be able to estimate the rate constants for the reactions between hydroxyl radicals and the individual boron species, eq 6 was used employing a linear combination of the contributions from all boron species on the basis of the pH-dependent speciation. This can be done since for each pH in the range of 5.5–11, the relative concentrations of the five boric acid species are unique, see Figure 4. By experimentally determining [*P*]_i_/[*P*]_ref_ at different pH and combining these results with the calculated speciation at the pH values for the respective experiments, we can obtain the individual rate constants from multilinear regression. The results of the fit can be seen in Figure 4 overlaying the boron fraction diagram and the experimental data for [*P*]_i_/[*P*]_ref_. The corresponding rate constants are listed in Table 1.

**Table 1:** Calculated Rate Constants for the Reactions of Hydroxyl Radicals with Five Boric Acid Species

*k* (M^–1^·s^–1^)	B(OH)_3_	B(OH)_4_^–^	B_2_(OH)_7_^–^	B_3_O_3_(OH)_4_^–^	B_4_O_5_(OH)_4_^2–^
from experimental fit	---	1.1 ± 0.7 × 10^6^	6.4 × 10^6^	---	6.8 × 10^6^
literature	<5 × 10^4^ (^22^)	<1 × 10^6^ (^26^)			

Under the conditions used in the experiments, only the borate ion, diborate, and tetraborate display significant reactivity toward the hydroxyl radical. Diborate and tetraborate are present in the same pH range, but at different concentrations, the highest concentration for both is reached at around pH 9.5. The estimated rate constants for the two species are very similar, 6.4 and 6.8 × 10^6^ M^–1^·s^–1^ for diborate and tetraborate, respectively. Triborate, which starts to form around pH 5.7 appears to be nonreactive toward the hydroxyl radical. Using 300 mM boric acid and 0.05 mM 3CCA at pH < 6 was not sufficient to obtain observable competition between the two possible reactions. Interestingly, the rate constant determined for the borate ion, 1.1 ± 0.7 × 10^6^ M^–1^·s^–1^, is very much in line with what was previously predicted to be the upper limit.^26^ Uncertainties of the rate constants are calculated as the standard deviation of the fitted rate constants from the multilinear regression. The uncertainty in the rate constants determined for the polyborate species is larger than that for the borate ion since several species are present at the same time at every pH, when diborate forms, tetraborate is also formed. The pH range where diborate and tetraborate are formed is the same and the shape of the speciation curves in the fraction diagram, Figure 4 is also very similar for the two species with a maximum concentration around pH 9.5. As a consequence, the concentration ratio between these two species is virtually unaffected by pH and it is impossible to determine the individual rate constants from multilinear regression with a reasonable uncertainty.

The model is unable to account for the two last experimental points above pH 11 where the ratio is lower than that expected from the kinetics. At a high pH, only the borate ion is present, but its reactivity cannot explain the sharp decrease. However, the hydroxyl radical has a p*K*_a_ of 11.9,^40^ and forms the oxygen radical anion, O^–^ upon deprotonation. The oxygen anion radical is nucleophilic, while the hydroxyl radical is electrophilic. In general, the oxygen radical anion displays lower reactivity than the hydroxyl radical and it is quite likely that the isomeric product distribution upon reaction with aromatic substances will differ significantly between the two radicals.^41^ For hydroxylation of unsubstituted coumarin, the rate constant is decreased from 6.4 × 10^9^ M^–1^·s^–1^ for the hydroxyl radical to 0.5 × 10^9^ M^–1^·s^–1^ for the oxygen radical anion.^42^ The addition of sodium hydroxide to increase the pH could also have an effect on the yield of 7OHCCA. This is because sodium hydroxide contains carbonate as an impurity. The carbonate ion and bicarbonate are reactive toward the hydroxyl radical, and at high concentrations, this could compete with 3CCA for the hydroxyl radical.^43^ For these reasons, the two points above pH 11 are not included in the fitting; the model is only fitted to the first 12 points in the pH interval of 4–11.

As the experimental conditions used above were not sufficient to observe any appreciable reactivity of the boric acid, we decided to increase the concentration ratio by increasing the concentration of boric acid to 600 mM and decreasing the concentration of 3CCA to 0.025 mM. To avoid large relative changes in 3CCA concentration during the experiment, the total irradiation time was reduced to 4 min. No boric acid was added to the reference system. The pH in the system containing 600 mM boric acid was adjusted with NaOH from 3.9 to 4.7 which was the pH in the system without boric acid. With the halved coumarin concentration and doubled boric acid concentration, the sensitivity is increased four times.

**Figure 5:**
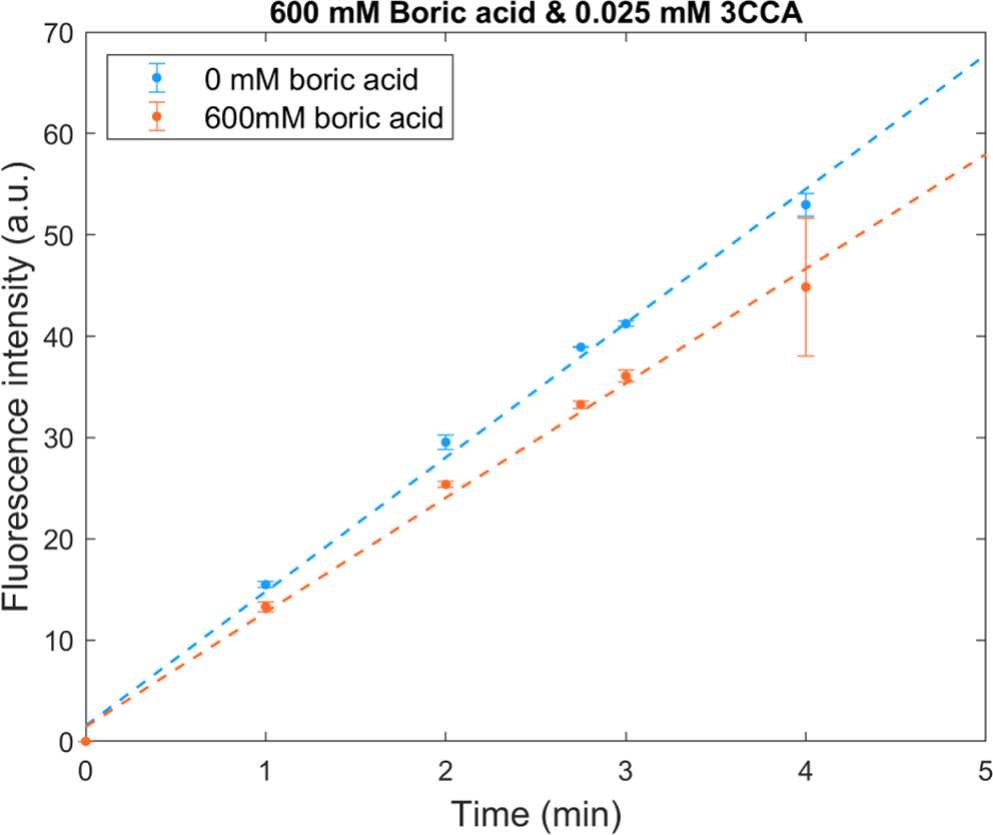
Fluorescence intensity for 7OHCCA for irradiation times up to 4 min. Both solutions contained 0.025 mM 3CCA and one of them had a boric acid concentration of 600 mM. pH of both solutions was 4.7. The slopes are 13.24 ± 0.23 and 11.30 ± 0.49 for the solution containing 0 and 600 mM boric acid, respectively.

Due to the reduced irradiation time from 8 to 4 min, the amount of sample added to the cuvette was increased from 25 to 50 μL to maintain a measurable signal. The sample was still diluted to 2.5 mL using 100 mM tetraborate buffer. In Figure 5, the yield of 7OHCCA can be seen for the two systems, the reference system contains only 3CCA and the second system contains both boric acid and 3CCA. The slopes are 13.24 ± 0.23 and 11.30 ± 0.49 for the boric acid-free system and the boric acid system, respectively. When 600 mM boric acid is present, the yield of 7OHCCA is decreased by 15% compared to the boric acid-free system, and the [*P*]_i_/[*P*]_ref_ ratio is 0.85. The corresponding rate constant estimated from eq 6 is 3.6 ± 1.3 × 10^4^ M^–1^·s^–1^. This is in line with the previously determined upper limit of 5 × 10^4^ M^–1^·s^–1^.^22^ The error bars in Figure 5 are based on the standard deviation for four measurements, except for the points at 2.75 and 3 min, which are based on two measurements.

The borate ion has been considered to be nonreactive which is clearly not the case.^26,44^ The diborate ion, which is the dominant polyborate species above 200 °C, is also reactive toward the hydroxyl radical, possibly even more so than the borate ion with a rate constant of 6.4 × 10^6^ M^–1^·s^–1^ compared to 1.1 × 10^6^ M^–1^·s^–1^ at room temperature. The rate constants are important for a model of the reactor chemistry in a reactor that uses boric acid for reactivity control. The reaction of hydroxyl radicals with boron species can affect hydrogen levels within a reactor which was previously seen when investigating the reactivity of boric acid.^23^ The reactive boron species can scavenge the hydroxyl radicals and stop the reaction between hydrogen and the hydroxyl radical which has a rate constant of 4.3 × 10^7^ M^–1^·s^–1^.^45^ The Guidelines on primary water chemistry from EPRI states that the dissolved hydrogen level should be higher than 25 cm^3^ STP per kg of water during operation.^6^ This corresponds to 0.8 mM hydrogen at 300 °C. Using the rate constants determined and speciation for 30 mM boron at pH 7, the amount of hydroxyl radicals that react with boric acid species will be roughly 4.5% of that for the reaction with hydrogen if the dissolved concentration is 0.8 mM. It should be pointed out that rate constants at room temperature and at 300 °C could differ significantly depending on the activation energy of the reactions.

The borate radical formed from the reaction between the borate ion and the hydroxyl radical has previously been investigated by Padmaja et al.^35,46,47^ Peroxydisulfate was used in a laser flash photolysis study to generate sulfate radicals which in turn reacted with the borate ion in the following reaction (eq 7). The properties of the formed borate radical were investigated, and its p*K*_a_ was determined to be 10.75. The reduction potential was estimated to be 1.4 V vs SHE from a comparison of the rate constant for the oxidation of phenolate with rate constants for other inorganic radicals with known reduction potential. The one-electron reduction potential is lower than that of the hydroxyl radical at high pH, 1.9 V vs SHE.^48^ By investigating the reactivity of the radical toward the benzoate ion at different ionic strengths, Padmaja et al. could establish that the charge of the deprotonated borate radical was negative one at pH 11.5. The structure with the lowest energy of the protonated borate radical was calculated by Bhattacharyya and Malar to be of *C*_2v_ symmetry. For the deprotonated borate radical B(OH)3O^•–^, the difference in energy between tetrahedral *T*_d_ and *C*_3v_ structure was calculated to be lower than 5 kcal/mol.^49^
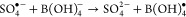
7

The borate anion is expected to react in a similar way to the hydroxyl radical. For the hydroxyl radical, the reaction could be either electron transfer or hydrogen abstraction. To yield the same product in the reaction with boric acid, the hydroxyl radical must add boric acid; see eq 8. Addition of an electrophilic radical, such as the hydroxyl radical, to an electron-poor boron atom is not expected to be very fast.
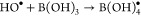
8

In a recently published PhD thesis, the rate constant for the reaction between the hydroxyl radical and the borate ion was determined using pulse radiolysis to be 2.24 × 10^6^ M^–1^·s^–1^.^50^ The rate constant is in the same range as reported here, 1.1 × 10^6^ M^–1^·s^–1^. Interestingly, the Gibbs free energies of the reaction between the hydroxyl radical and boric acid and the reaction between the hydroxyl radical and borate are also presented in the same thesis. These energies are based on DFT calculations. The resulting free energies are + 50 and −36.9 kJ/mol for the reaction with boric acid and borate, respectively. This implies that the reaction with borate is spontaneous, while the reaction with boric acid is not. In very qualitative terms, this trend is in line with our experimental observations.

## Conclusions

The system with 0.05 mM coumarin-3-carboxylic acid and 300 mM boric acid showed good competition at pH over 7, where the counter-base borate and the two polyanion species diborate and tetraborate started to form. The rate constants are 1.1 × 10^6^, 6.4 × 10^6^, and 6.8 × 10^6^ M^–1^·s^–1^ for borate, diborate, and tetraborate, respectively. No reactivity of triborate was observed. The rate constant for the reaction between the hydroxyl radical and boric acid was determined to be 3.6 × 10^4^ M^–1^·s^–1^. The rate constants for boric acid and the counter-base are consistent with previously estimated upper limits. When using borate buffer to adjust the pH in a system where hydroxyl radicals are generated, it should be taken into consideration that it is not competing with the reaction of interest. This can especially be a problem for reactions at high pH and high concentrations of borate when the reactivity of the hydroxyl radicals is expected to be low toward the scavenger.

## Supporting Information Available

Fluorescence excitation and emission spectra of 7-hydroxycoumarin-3-carboxylic acid and fluorescence intensity vs irradiation time for pH 4.5–11.5 (PDF)
