# Supplementary material for: The Reactivity of Hydroxyl Radicals toward Boric Acid as a Function of pH
Source: J Phys Chem A. 2024 Sep 3;128(36):7593–600. doi: 10.1021/acs.jpca.4c03933 (PMC11403659; doi:10.1021/acs.jpca.4c03933)
Supplement: Supplementary file 1 — jp4c03933_si_001.pdf [file jp4c03933_si_001.pdf]

Supporting information

The Reactivity of Hydroxyl Radicals Towards Boric Acid as Function of pH

Fredrik Petersson* and Mats Jonsson, Department of Chemistry, KTH Royal Institute of Technology, SE – 100 44 Stockholm, Sweden

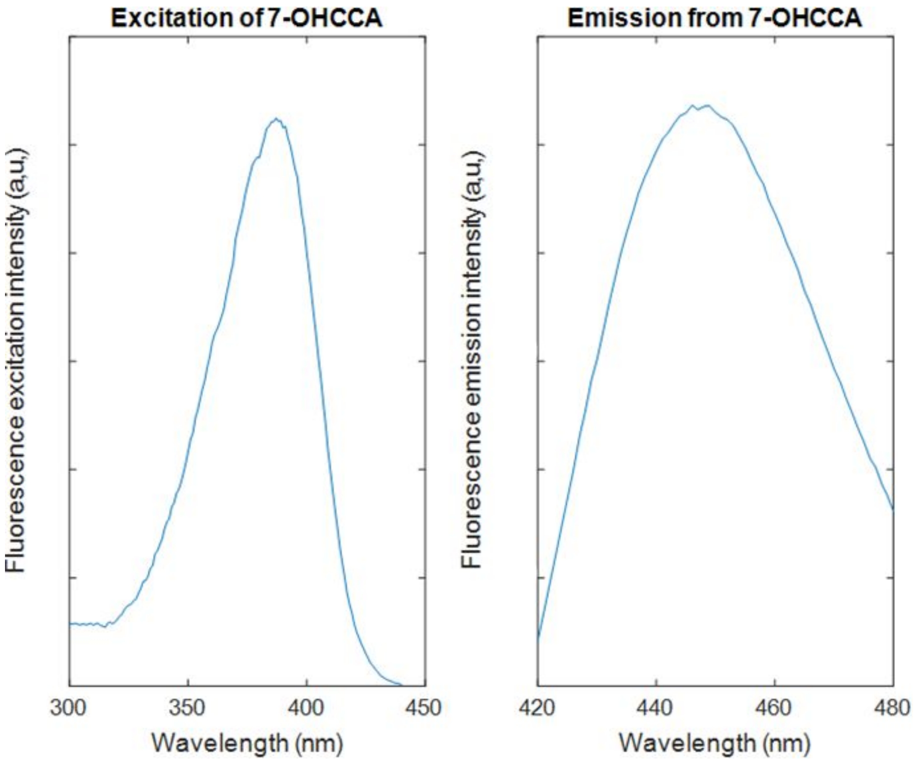

Figure S1. Fluorescence excitation and emission spectra of 7-hydroxycoumarin-3-carboxylic acid in 100 mM borate buffer at pH 9.23. For the excitation spectrum, the emission is recorded at 450 nm and for the excitation spectrum the excitation light had a wavelength of 385 nm.

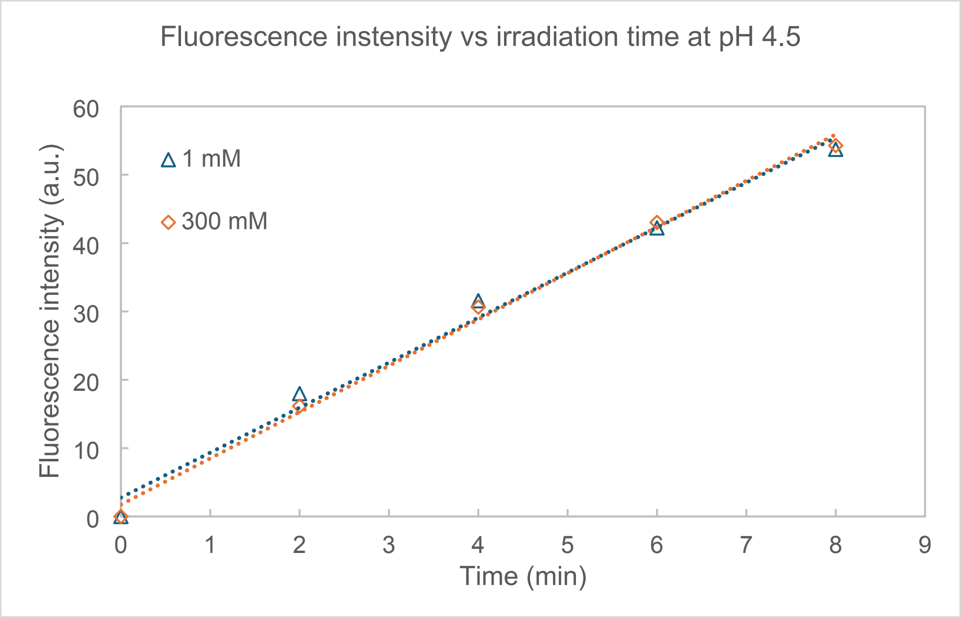

Figure S2. Fluorescence intensity vs irradiation time for the samples with a pH of 4.5 the slope of the lines is 6.58 ± 0.42 and 6.77 ± 0.29 for 1 mM and 300 mM respectively.

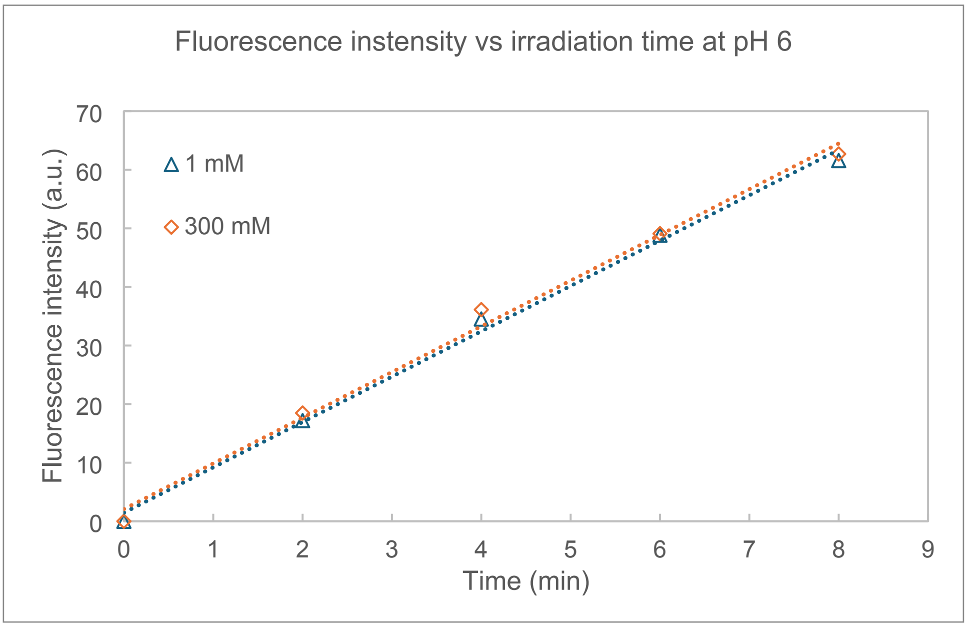

Figure S3. Fluorescence intensity vs irradiation time for the samples with a pH of 6 the slope of the lines is 7.74 ± 0.30 and 7.80 ± 0.37 for 1 mM and 300 mM respectively.

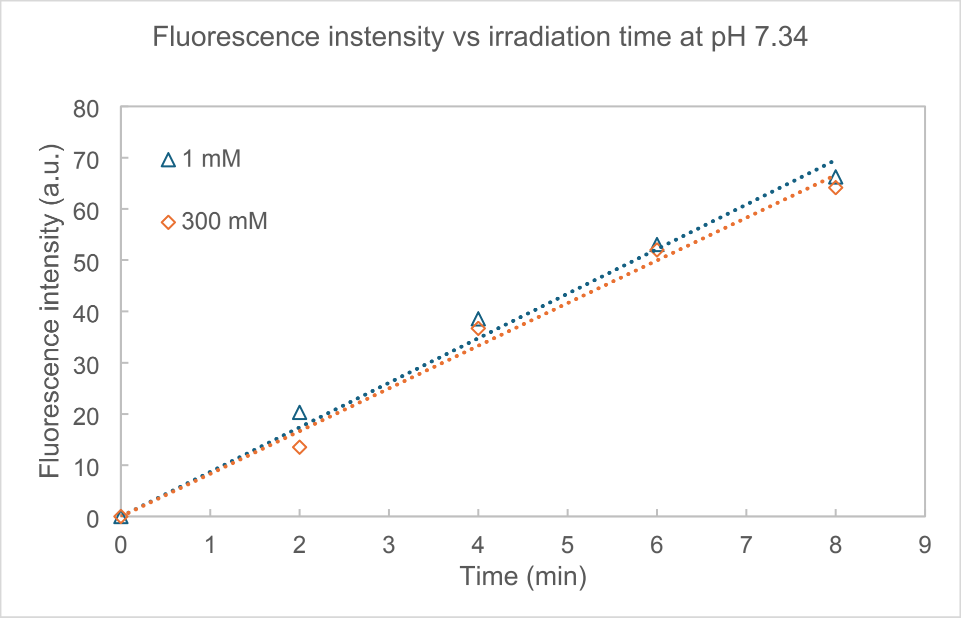

Figure S4. Fluorescence intensity vs irradiation time for the samples with a pH of 7.34 the slope of the lines is 8.69 ± 0.27 and 8.32 ± 0.26 for 1 mM and 300 mM respectively.

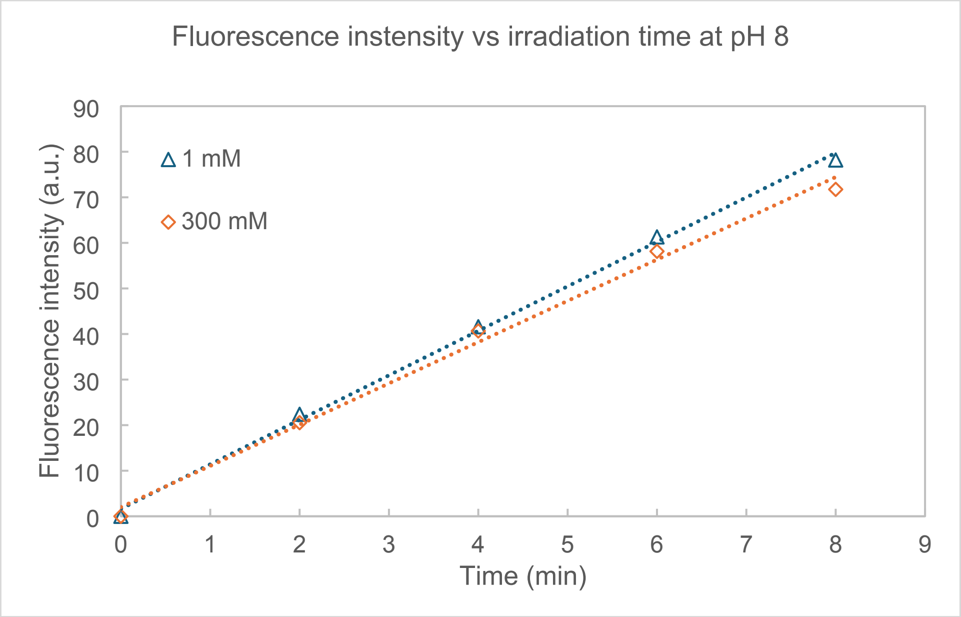

Figure S5 Fluorescence intensity vs irradiation time for the samples with a pH of 8 the slope of the lines is 9.77 ± 0.27 and 9.05 ± 0.42 for 1 mM and 300 mM respectively.

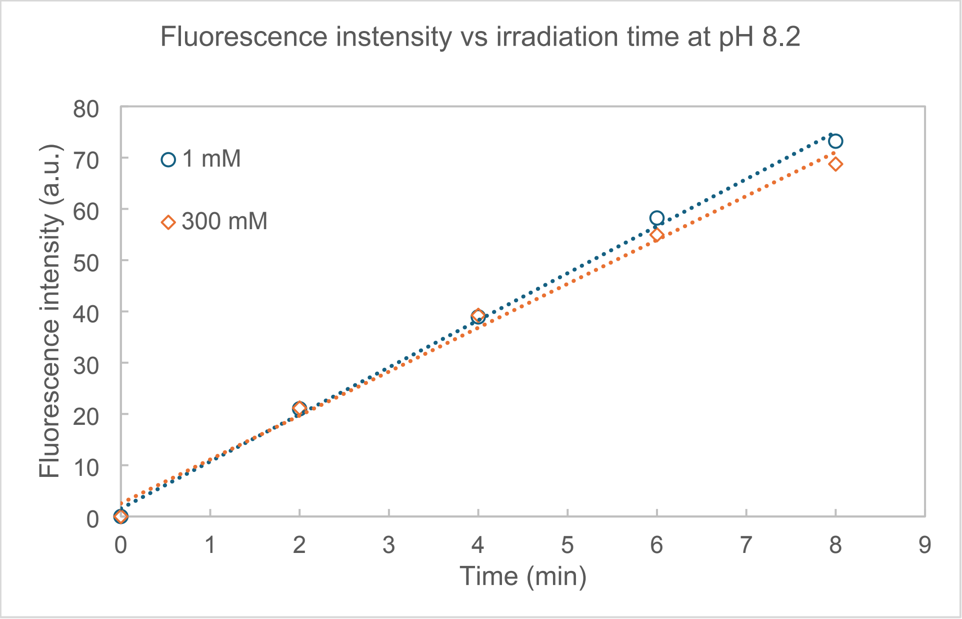

Figure S6. Fluorescence intensity vs irradiation time for the samples with a pH of 8.2 the slope of the lines is 9.18± 0.29 and 8.56 ± 0.42 for 1 mM and 300 mM respectively.

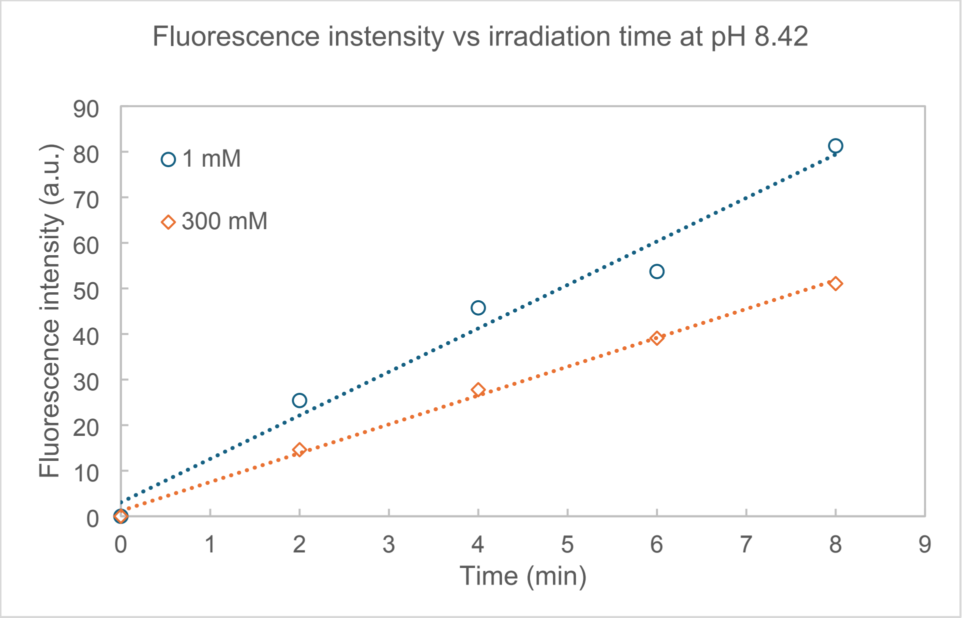

Figure S7. Fluorescence intensity vs irradiation time for the samples with a pH of 8.42 the slope of the lines is 9.54 ± 0.85 and 6.33 ± 0.19 for 1 mM and 300 mM respectively.

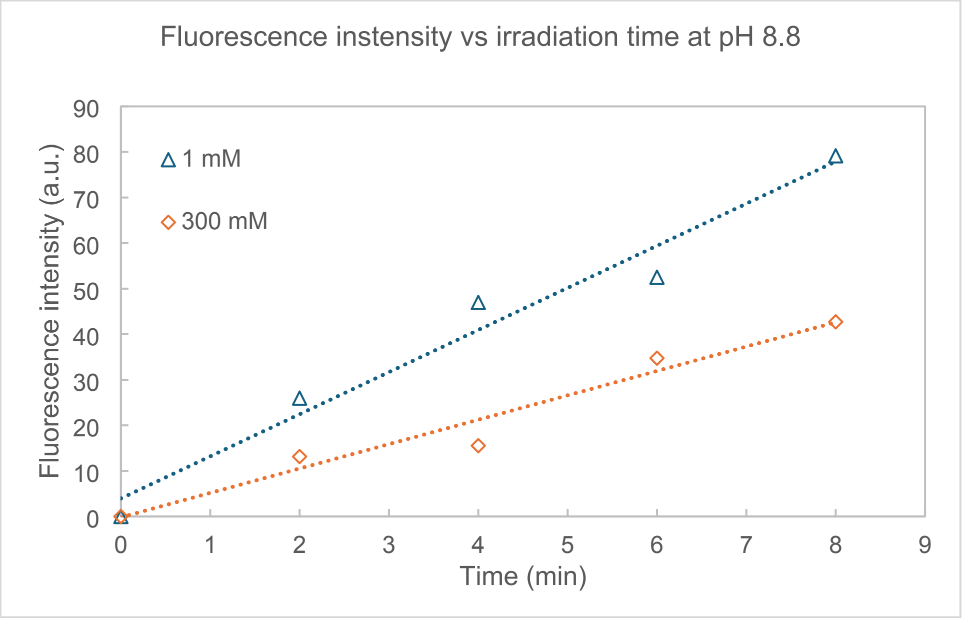

Figure S8. Fluorescence intensity vs irradiation time for the samples with a pH of 8.8 the slope of the lines is 9.24 ± 0.97 and 5.35 ± 0.63 for 1 mM and 300 mM respectively.

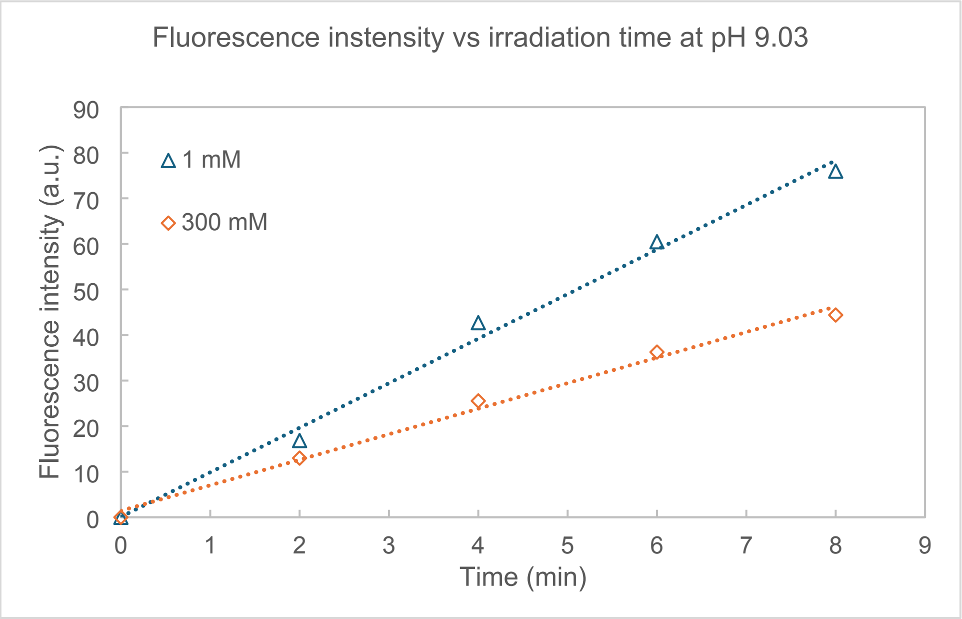

Figure S9. Fluorescence intensity vs irradiation time for the samples with a pH of 9.03 the slope of the lines is 9.78 ± 0.49 and 5.60 ± 0.29 for 1 mM and 300 mM respectively.

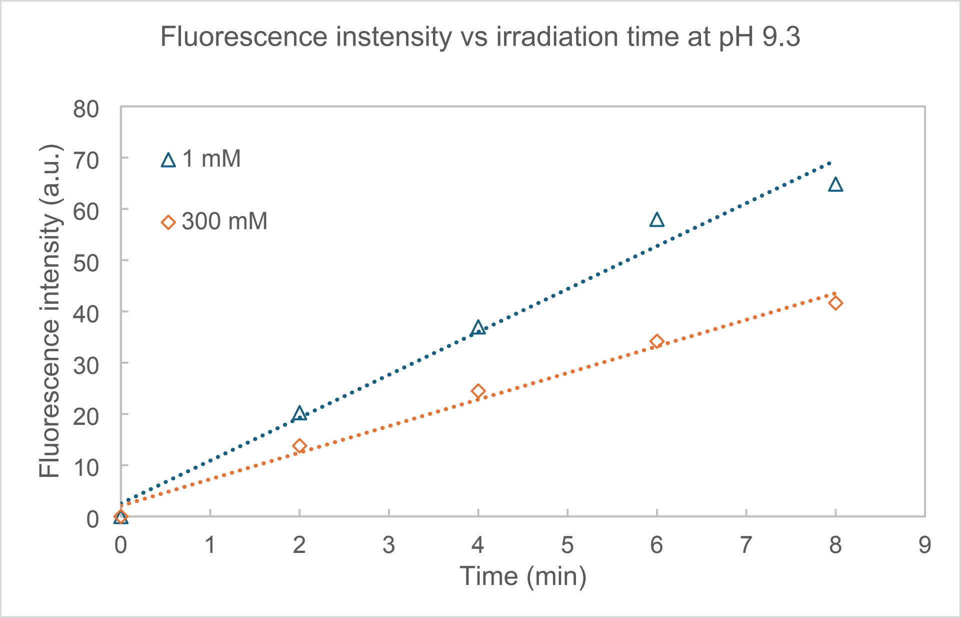

Figure S10. Fluorescence intensity vs irradiation time for the samples with a pH of 9.3 the slope of the lines is 8.37 ± 0.69 and 5.18 ± 0.34 for 1 mM and 300 mM respectively.

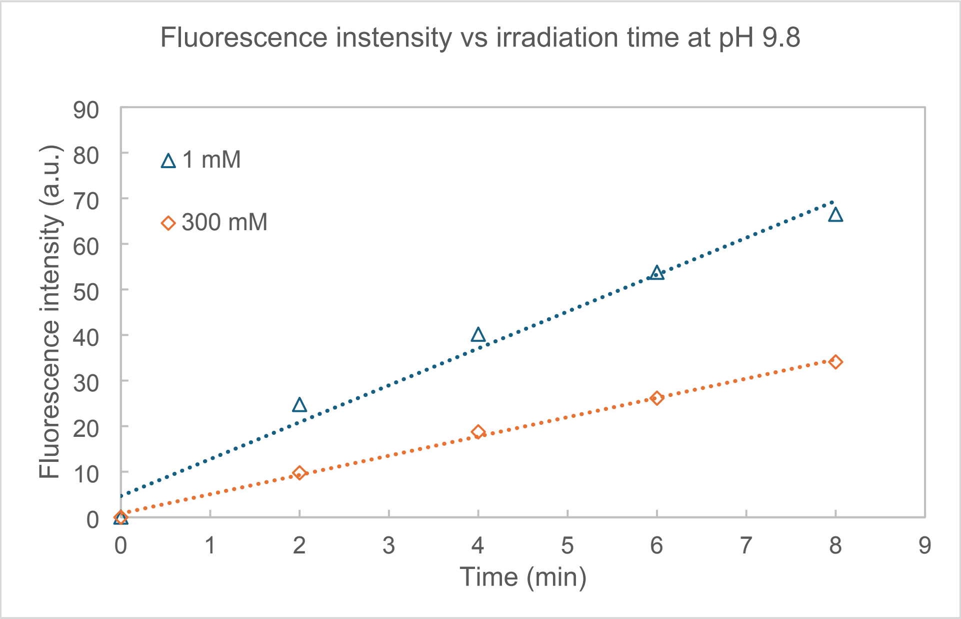

Figure S11. Fluorescence intensity vs irradiation time for the samples with a pH of 9.8 the slope of the lines is 8.10 ± 0.68 and 4.23 ± 0.13 for 1 mM and 300 mM respectively.

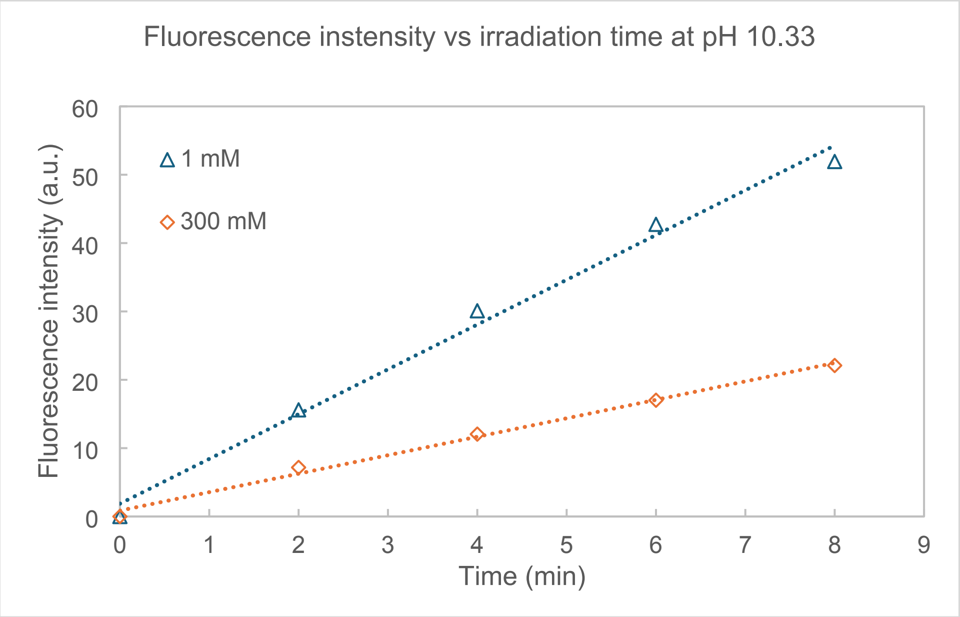

Figure S12. Fluorescence intensity vs irradiation time for the samples with a pH of 10.33 the slope of the lines is 6.55 ± 0.36 and 2.70 ± 0.12 for 1 mM and 300 mM respectively.

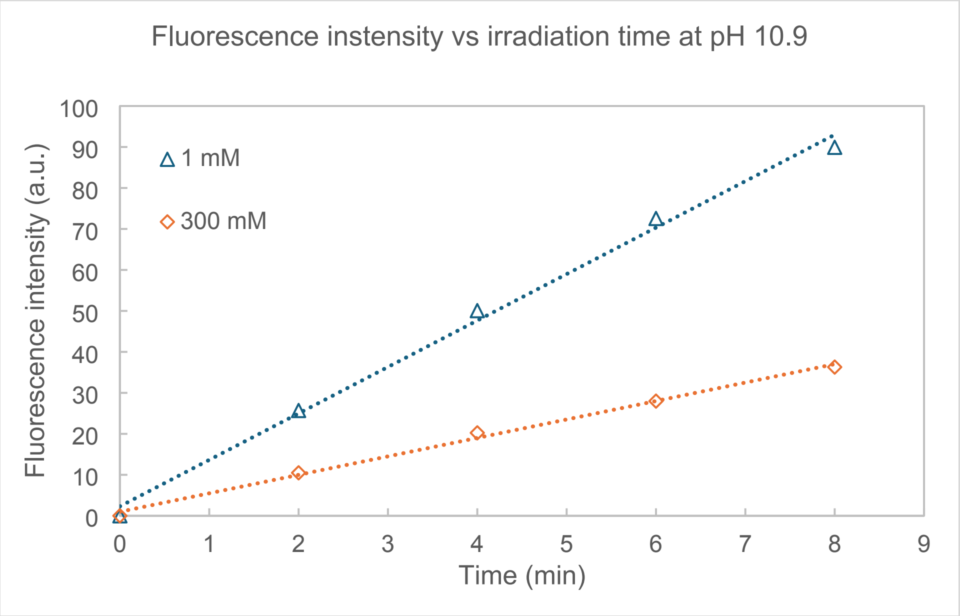

Figure S13. Fluorescence intensity vs irradiation time for the samples with a pH of 10.9 the slope of the lines is 11.35 ± 0.46 and 4.50 ± 0.16 for 1 mM and 300 mM respectively.

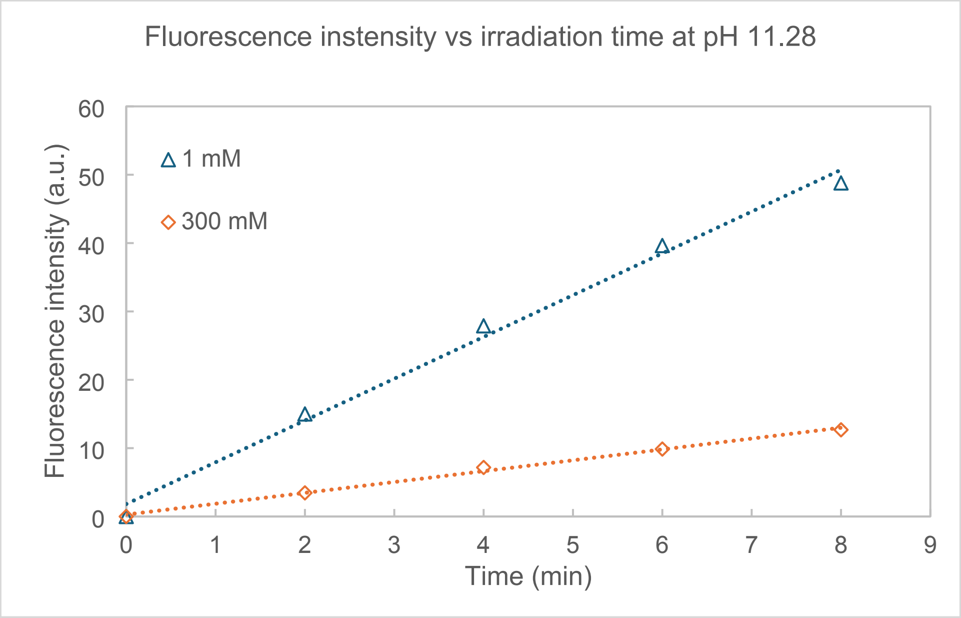

Figure S14. Fluorescence intensity vs irradiation time for the samples with a pH of 11.28 the slope of the lines is 6.11 ± 0.31 and 1.59 ± 0.06 for 1 mM and 300 mM respectively.

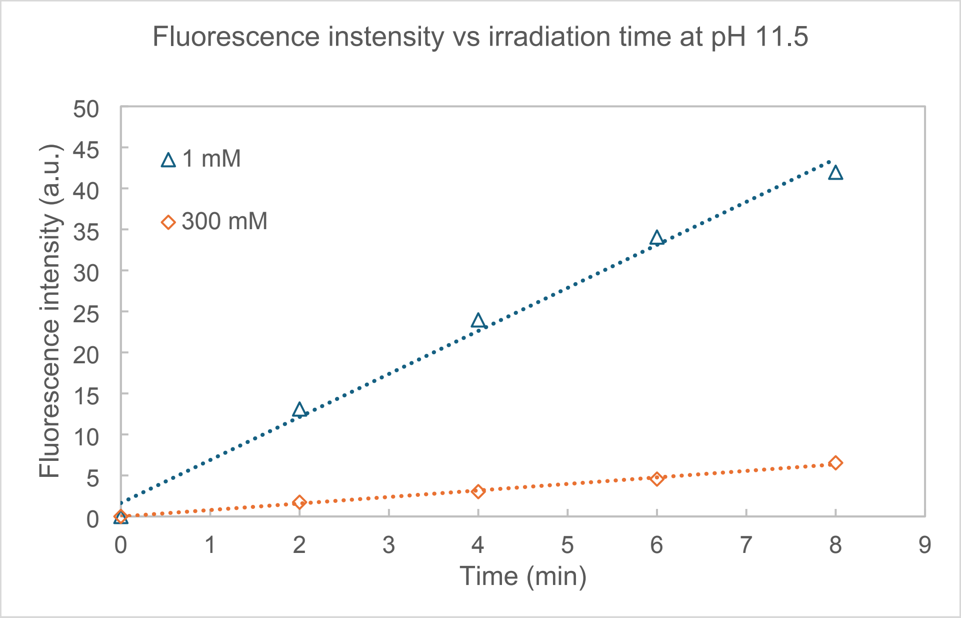

Figure S15 Fluorescence intensity vs irradiation time for the samples with a pH of 11.5 the slope of the lines is 5.24 ± 0.27 and 0.80 ± 0.03 for 1 mM and 300 mM respectively.

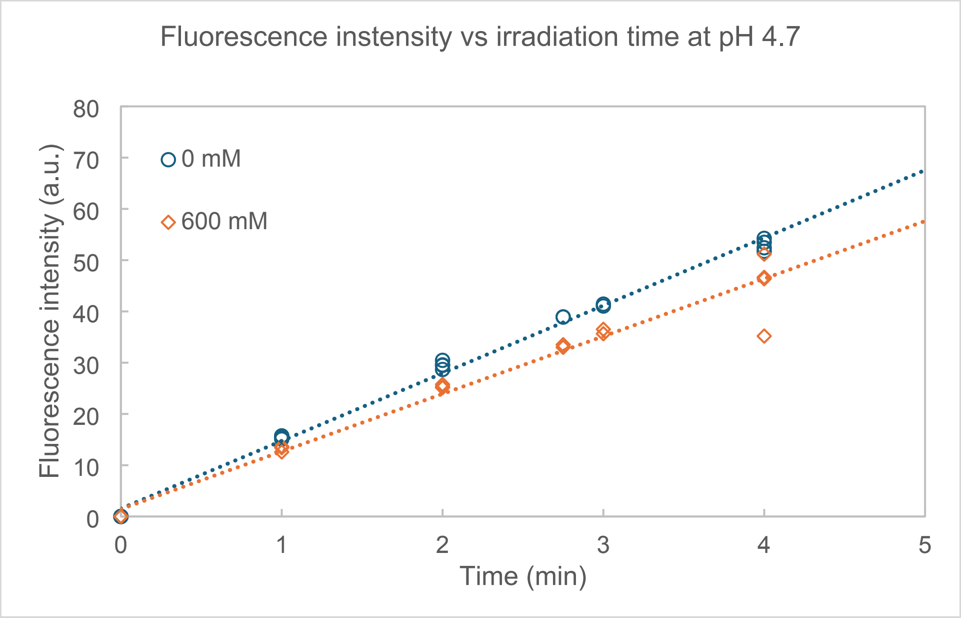

Figure S16 Fluorescence intensity vs irradiation time for the samples with 0 mM boric acid and 600 mM boric acid. The pH during irradiation was 4.7 and the slope of the lines are 13.24 ± 0.23 and 11.30 ± 0.49 for the solution containing 0 mM boric acid and 600 mM boric acid respectively.
